# Development and Validation of an IDH1-Associated Immune Prognostic Signature for Diffuse Lower-Grade Glioma

**DOI:** 10.3389/fonc.2019.01310

**Published:** 2019-11-22

**Authors:** Xiangyang Deng, Dongdong Lin, Bo Chen, Xiaojia Zhang, Xingxing Xu, Zelin Yang, Xuchao Shen, Liang Yang, Xiangqi Lu, Hansong Sheng, Bo Yin, Nu Zhang, Jian Lin

**Affiliations:** ^1^Department of Neurosurgery, The Second Affiliated Hospital and Yuying Children's Hospital of Wenzhou Medical University, Wenzhou, China; ^2^The Second Clinical Medical College, Wenzhou Medical University, Wenzhou, China; ^3^School of Basic Medical Sciences, Wenzhou Medical University, Wenzhou, China

**Keywords:** lower-grade glioma, IDH1, mutation, immune prognostic signature, nomogram

## Abstract

A mutation in the isocitrate dehydrogenase 1 (IDH1) gene is the most common mutation in diffuse lower-grade gliomas (LGGs), and it is significantly related to the prognosis of LGGs. We aimed to explore the influence of the IDH1 mutation on the immune microenvironment and develop an IDH1-associated immune prognostic signature (IPS) for predicting prognosis in LGGs. IDH1 mutation status and RNA expression were investigated in two different public cohorts. To develop an IPS, LASSO Cox analysis was conducted for immune-related genes that were differentially expressed between IDH1^wt^ and IDH1^mut^ LGG patients. Then, we systematically analyzed the influence of the IPS on the immune microenvironment. A total of 41 immune prognostic genes were identified based on the IDH1 mutation status. A four-gene IPS was established and LGG patients were effectively stratified into low- and high-risk groups in both the training and validation sets. Stratification analysis and multivariate Cox analysis revealed that the IPS was an independent prognostic factor. We also found that high-risk LGG patients had higher levels of infiltrating B cells, CD4+ T cells, CD8+ T cells, neutrophils, macrophages and dendritic cells, and expressed higher levels of CTLA-4, PD-1 and TIM-3. Moreover, a novel nomogram model was established to estimate the overall survival in LGG patients. The current study provides novel insights into the LGG immune microenvironment and potential immunotherapies. The proposed IPS is a clinically promising biomarker that can be used to classify LGG patients into subgroups with distinct outcomes and immunophenotypes, with the potential to facilitate individualized management and improve prognosis.

## Introduction

Gliomas are the most commonly occurring type of malignant primary tumor of the central nervous system, which arise from astrocytic, oligodendroglial, mixed oligoastrocytic, or neuronal-glial cells, and result in significant morbidity and mortality (1, 2). According to the WHO classification system based on the histological type, diffuse lower-grade gliomas (LGGs) have a grade of II or III (3). Despite diverse natural course of this heterogeneous group, most LGGs will gradually evolve into higher-grade gliomas and eventually lead to death (4).

Some studies have indicated that key components of the immune response were significantly altered in gliomas, and subsequently led to immune evasion of tumors (5, 6). In addition to conventional treatment methods including surgery, radiotherapy and chemotherapy, immunotherapy is rapidly emerging as a promising treatment modality and works by evoking an anti-tumor immune response that inhibits immune evasion by the tumor. A number of immune-related parameters have been discovered to predict the outcomes of LGG patients (7, 8). However, there is still a lack of reliable biomarkers that can identify subsets of patients with potential sensitivity to immunotherapy. Moreover, few studies have systematically explored the immune microenvironment of LGG.

Based on the molecular profiles of gliomas, the mutation in the isocitrate dehydrogenase 1 (IDH1) gene has been identified to facilitate patient stratification and predict prognosis, along with other molecular markers including the 1p/19q co-deletion, methylguanine methyltransferase (MGMT) promoter methylation, tumor protein (TP) 53, and telomerase reverse transcriptase (TERT) promoters (9, 10). IDH1 encodes the cytosolic isocitrate dehydrogenase 1, an enzyme that catalyzes the oxidative decarboxylation of isocitrate to α-ketoglutarate and plays a critical role in cellular protection from oxidative stress (11, 12). Further studies have found this mutation to be present in up to 80% of LGG patients and was virtually absent in primary glioblastomas (13). More notably, research increasingly suggests that the IDH1 mutation conferred an immunologically quiescent phenotype (14–17). Berghoff et al. reported that the immunological tumor microenvironment was associated with IDH mutation status in gliomas. They found that IDH-mutant gliomas exhibit fewer tumor infiltrating lymphocytes (TILs) and show reduced expression of programmed death ligand 1 (PD-L1) protein compared to that in the wild-type counterparts, which may be at least in part due to differential PD-L1 gene promoter methylation levels (15). Bunse et al. also demonstrated that IDH-mutant gliomas display reduced T cell abundance and altered calcium signaling (17). Hence, we performed a comprehensive analysis to further explore the relationship between IDH1 mutation status and the immune response based on RNA sequencing (RNA-seq) data.

In the present study, we downloaded RNA-seq data from The Cancer Genome Atlas (TCGA) as a training set and from the Chinese Glioma Genome Atlas (CGGA) as a validation set. We systematically analyzed the influence of the IDH1 mutation on the immune microenvironment, and developed an immune prognostic signature (IPS) based on four IDH1-associated immune genes to classify patients into subgroups with distinct prognosis and immunophenotypes. We ascertained an independent role of this four-gene IPS and highlighted the potential value of the included genes to serve as therapeutic biomarkers. Furthermore, a reliable predictive nomogram model was designed to estimate overall survival (OS) for LGG patients.

## Materials and Methods

### Gene Expression Datasets and Immune-Related Genes

The RNA-seq data of 511 LGG samples were obtained from the TCGA database as a training set. Information regarding the somatic mutation status and clinical dataset of the corresponding LGG patients were also downloaded from the TCGA website (https://portal.gdc.cancer.gov/repository). From the CGGA dataset (http://www.cgga.org.cn/), we downloaded RNA-seq data of 172 LGG samples as a validation set. In addition, a comprehensive immune-related gene set, identified to actively participate in the process of immune activity, was extracted from the Immunology Database and Analysis Portal (ImmPort) database (https://immport.niaid.nih.gov) (18). This was used to identify immune genes that were differentially expressed between patients with (IDH1^mut^) and without IDH1 mutation (IDH1^wt^).

### Differential Expression Analysis

Differential expression analysis was conducted using the “DESeq2” R package (19). The log2 |fold change| > 1.5 and adj. *P* < 0.05 were set as the cut-off values to screen for differentially expressed genes.

### Functional Enrichment Analysis

Metascape (http://metascape.Org) was used to perform functional and pathway enrichment analyses to explore the potential molecular mechanisms of the selected genes (20). Functional enrichment was conducted for Gene Ontology (GO) terms including the cellular component, biological process, and molecular function categories. The Kyoto Encyclopedia of Genes and Genomes (KEGG) pathways were also enriched. Only terms with a *P* < 0.01 and the number of enriched genes ≥3 were considered as significant and grouped into clusters based on their membership similarities. The most enriched term within a cluster was selected as the one to represent the cluster.

### Construction of the Immune Prognostic Signature

Following quality filtering to exclude patients with missing survival information or a survival time of 0 days, there were 506 samples subjected to subsequent analysis. For further analysis, the transcriptome profiling of RNA measured by FPKM values was performed using the log2-based transformation. On the basis of the differentially expressed immune genes (DEIGs), Kaplan-Meier analysis was first performed to screen for prognostic genes in the TCGA set. These genes which were validated in CGGA were put into the Cox regression model with least absolute shrinkage and selection operator (LASSO) penalty for analysis using the “glmnet” R package (21–23). Finally, an IPS was constructed by weighting the Cox regression coefficients to calculate a risk score for each patient. Based on the optimal cut-off values obtained by the “survminer” R package, LGG patients were classified as low- and high-risk according to their risk score. To appraise the prognostic performance of the IPS, Kaplan-Meier analysis and the log-rank test were employed. Time-dependent receiver operating characteristic (ROC) curves were depicted to evaluate the sensitivity and specificity using the “timeROC” R package (24). Area under the curve (AUC) values were calculated from the ROC curves.

### Principal Components Analysis (PCA) and Gene Set Enrichment Analysis (GSEA)

PCA was carried out using the “pca3d” R package to investigate gene expression patterns of grouped patients. GSEA (http://www.broadinstitute.org/gsea/index.jsp) was conducted between high- and low-risk phenotypes (25). A nominal *P* < 0.05 and a false discovery rate (FDR) < 0.25 were considered statistically significant.

### TIMER Database Analysis

The TIMER database (https://cistrome.shinyapps.io/timer) is a comprehensive resource to analyze and visualize immune infiltrates among different cancer types (26). TIMER reanalyzes gene expression profiles, which includes 10,897 samples across 32 cancer types from TCGA to estimate six immune cell types in the tumor microenvironment, including B cells, CD4+ T cells, CD8+ T cells, macrophages, neutrophils, and dendritic cells (26). The data of immune infiltrate levels of LGG patients was extracted from the TIMER database to investigate the association with the IPS.

### Development and Validation of the Nomogram

Univariate and multivariate Cox analyses were performed to assess the independent prognostic ability of the IPS. Then, a novel nomogram was generated based on the results of the multivariate Cox analysis using the “rms” R package and externally validated in the CGGA cohort. We conducted 1-, 3-, 5-year OS calibrations to determine the predictive accuracy of the nomogram model. The concordance index (C-index) was used to evaluate the discrimination of the model. Bootstraps with 1,000 resamples were calculated to correct the C-index (27). In addition, the time-dependent ROC curves were plotted to illustrate the predictive performance. To assess the clinical utility of the nomogram, decision curve analysis (DCA) was employed to compare the benefits of different models.

### Statistical Analysis

Heatmaps were generated using the “pheatmap” R package. A volcano plot and violin plots were generated using the “ggplot2” R package. OS was defined as the primary outcome. Statistical analyses of this study were conducted using the R software (version 3.5.2), GraphPad Prism (version 7.0.0), and SPSS software (version 24.0). A two-sided *P* < 0.05 was regarded as significant.

## Results

### Identification of Differentially Expressed Immune Genes

**Figure 1:**
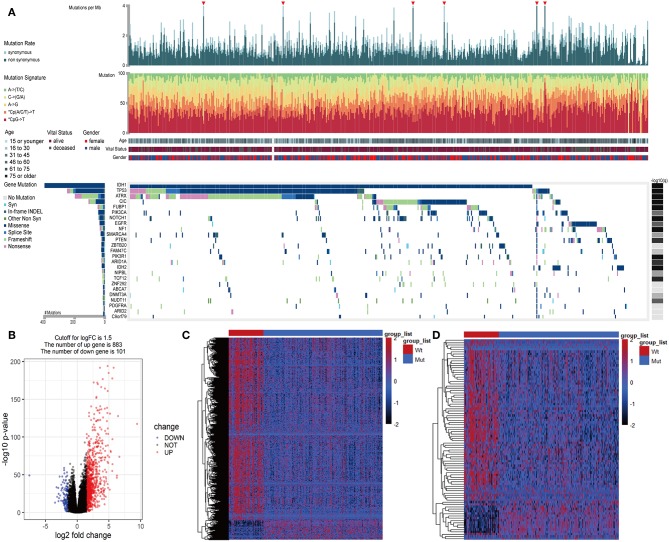
Identified IDH1-associated immune genes. **(A)** Genomic landscape of LGG and the mutational signatures in the TCGA dataset, which were assayed on the FireBrowse platform. **(B)** Volcano plot of 984 genes differentially expressed between IDH1^wt^ and IDH1^mut^ patients. **(C)** Heatmap of genes differentially expressed between IDH1^wt^ and IDH1^mut^ patients. **(D)** Heatmap of immune genes differentially expressed between IDH1^wt^ and IDH1^mut^ patients.

**Figure 2:**
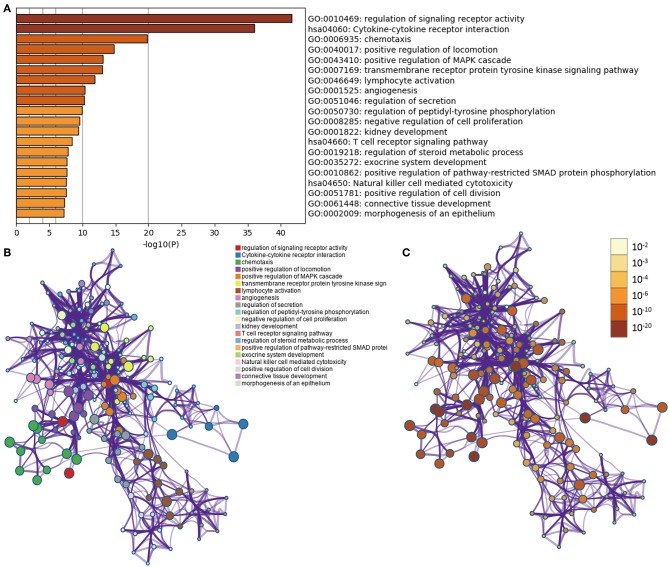
Functional analysis of 88 IDH1-associated immune genes. **(A)** Heatmap of enriched terms across input gene lists, colored by *P*-values. Network of enriched terms: **(B)** colored by cluster ID, where nodes that share the same cluster ID are typically close to each other; **(C)** colored by *p*-value, where terms containing more genes tend to have a more significant *P*-value.

In LGGs, the IDH1 mutation is the most common type of mutation (Figure 1A). Based on the DESeq2 algorithm, there were 984 genes identified that were differentially expressed between IDH1^wt^ and IDH1^mut^ patients, including 883 up-regulated and 101 down-regulated genes (Figures 1B,C). From this set of genes, 88 DEIGs were selected by the ImmPort database for further analysis (Figure 1D). As shown in Figures 2A–C, the DEIGs were mainly enriched in regulation of signaling receptor activity, chemotaxis, positive regulation of MAPK cascade, transmembrane receptor protein tyrosine kinase signaling pathway, lymphocyte activation (GO), and cytokine-cytokine receptor interaction (KEGG).

### Construction of the Immune Prognostic Signature

**Figure 3:**
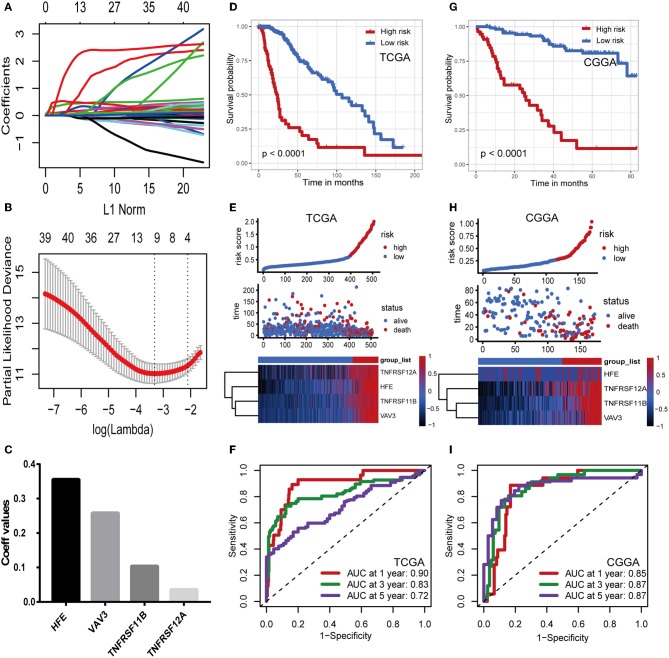
Construction and validation of the immune prognostic signature. **(A,B)** LASSO Cox analysis identified four genes most correlated to overall survival in TCGA set. **(C)** Coefficient values for each of the four selected genes. **(D,G)** Kaplan–Meier curves of overall survival for LGG patients based on the IPS in TCGA cohort and CGGA cohort. **(E,H)** Risk scores distribution, survival status of each patient, and heatmaps of prognostic four-gene signature in TCGA and CGGA cohorts. **(F,I)** Time-dependent ROC curve analysis of the IPS.

Considering the differences in immune gene expression between IDH1^wt^ and IDH1^mut^ patients, we evaluated the prognostic value of DEIGs by Kaplan-Meier analysis. Log-rank tests were performed and revealed that 68 DEIGs were associated with prognosis. Using a cross validation with the CGGA set, 41 DEIGs were identified as showing significant correlation between gene expression and OS (Supplementary Table 1). Then, LASSO Cox analysis was performed to select genes with the best prognostic value and to build an IPS in the TCGA cohort (Figures 3A,B). Risk scores were calculated for each sample (risk score = 0.036^*^TNFRSF12A + 0.259^*^VAV3 + 0.104^*^TNFRSF11B + 0.356^*^HFE, Figure 3C). Patients in the TCGA cohort then were assigned to a high- or low-risk group using the optimal cut-off value obtained with the “survminer” R package. The Kaplan-Meier analysis demonstrated that patients with a high-risk score were correlated with worse outcomes (Figure 3D). Risk score distribution and gene expression patterns are shown in Figure 3E. The time-dependent ROC curve analysis of the IPS in the TCGA cohort indicated a promising prognostic ability for OS (1-year AUC = 0.90, 3-year AUC = 0.83, 5-year AUC = 0.72, Figure 3F).

### Validation of the Immune Prognostic Signature

To confirm that the IPS had a robust prognostic value, the same formula was applied to the CGGA set, which consisted of 172 LGG patients. Using the cut-off value obtained from the corresponding cohort, patients were divided into high- and low-risk groups. Consistent with the findings in the TCGA database, patients with high-risk scores had significantly worse OS than those with low-risk scores (Figure 3G). Risk score distribution and gene expression patterns are shown in Figure 3H. The time-dependent ROC analysis also showed that the IPS had high sensitivity and specificity (Figure 3I). AUC values were 0.85, 0.87, and 0.87 for 1-, 3-, and 5-year OS, revealing the high predictive value of the IPS for LGG patients.

### Stratification Analyses

**Figure 4:**
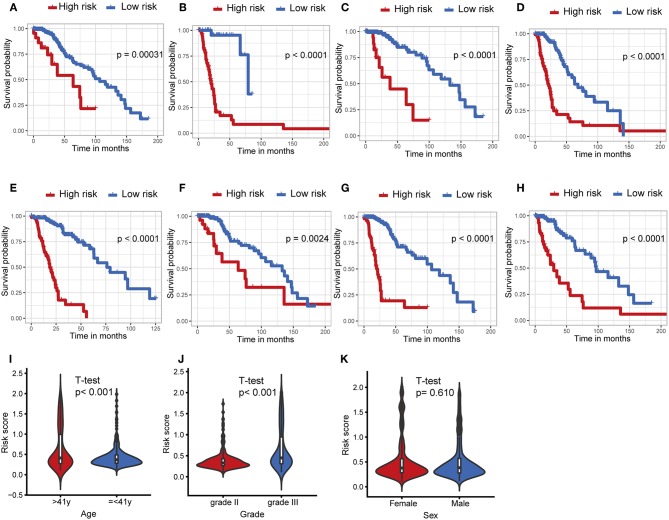
Stratification analysis. The Kaplan–Meier analysis of the IPS grouping according to patients with **(A)** IDH1 mutant, **(B)** IDH1 wildtype, **(C)** grade II, **(D)** grade III, **(E)** > 41 years, **(F)** ≤ 41 years, **(G)** male, and **(H)** female. The risk score was group by **(I)** age, tumor grade **(J)**, and **(K)** sex.

The IDH1 mutation is a stable marker for better prognosis in LGG. Stratification analyses were carried out to determine whether the predictive ability of the IPS would remain stable in distinct subgroups. As shown in Figures 4A,B, patients in the high-risk group showed worse survival compared to those in the low-risk group in both IDH1^wt^ and IDH1^mut^ subgroups. We also demonstrated that the IPS was still a powerful marker for predicting OS in patients with grade II or grade III tumors, younger or older, and male or female patients (Figures 4C–H).

Afterwards, we attempted to determine the statistical difference in the distribution of clinicopathological features between low- and high-risk groups. The risk scores distributed differently in stratified patients validating their association with the IPS. Patients with grade III tumor or at older ages exhibited a higher-risk level (Figures 4I,J). Whereas, there was no association between risk score and sex (Figure 4K).

### High Risk Indicated an Enhanced Local Immune Phenotype

**Figure 5:**
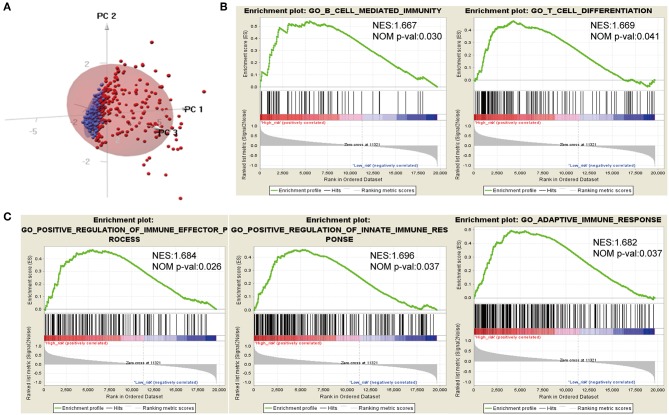
Different immune phenotypes between high- and low-risk groups in TCGA cohort. **(A)** Principal components analysis of IDH1-associated immune genes between high- and low-risk groups. Blue color indicates low-risk patients, and red color represents high-risk patients. **(B,C)** Gene set enrichment analysis for comparing immune phenotype between high- and low-risk groups. Significant enrichment of five immune-related GO terms in high-risk group. FDR, false discovery rate; NES, normalized enrichment score.

Considering different prognosis, we investigated differences between risk groups using RNA-seq data. Based on the genes comprising the IPS, PCA was performed and revealed that patients in high- or low-risk groups were distributed in discrete directions indicating differences in the immune phenotype (Figure 5A). GSEA was then conducted between the high- and low-risk groups, and more immune-related biological processes were found significantly enriched in the high-risk group, indicating that the high-risk score conferred an enhanced immune phenotype (Figures 5B,C, Supplementary Table 2).

### Timer Database Analysis and Immune Checkpoints Analysis

**Figure 6:**
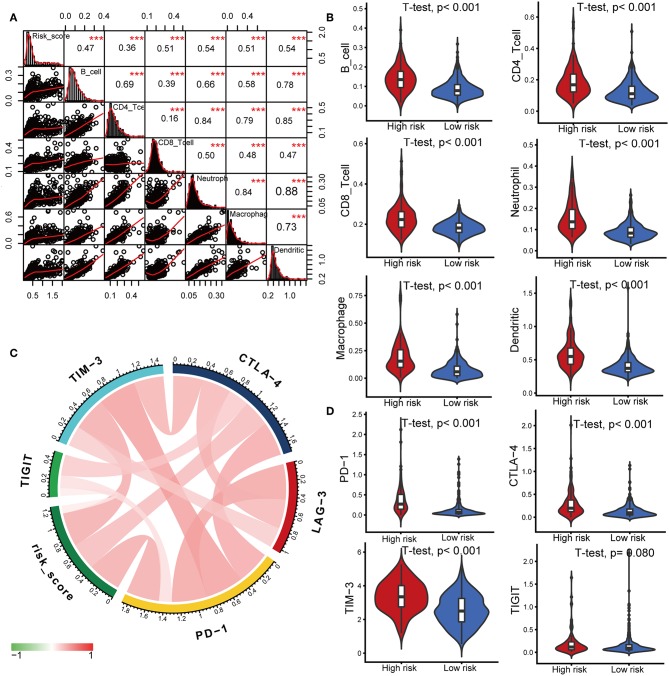
Correlations of the IPS with infiltrating immune cell proportions and immune checkpoints. **(A)** Correlation of the risk score with infiltrating immune cell proportions. Pearson's correlation coefficient values with the level of significance were shown on the top of the diagonal. ^***^P < 0.001. **(B)** Violin plots visualizing significantly different immune cell proportions between high- and low-risk patients. **(C)** Correlation of the risk score with the expression of several crucial immune checkpoints **(D)** Violin plots visualizing significantly different immune checkpoints between high- and low-risk patients.

Characterization of the immune infiltration landscape is important to explore the status of the immune microenvironment and investigate the tumor-immune interaction. We applied the TIMER tool to identify potential relationships between the IPS and infiltrating immune cells including B cells, CD4+ T cells, CD8+ T cells, neutrophils, macrophages and dendritic cells. As shown in Figure 6A, tumor-infiltrating immune cells were strongly interrelated and exhibited positive correlation with our IPS. Patients in the high-risk group had significantly higher proportions of infiltrating B cells, CD4+ T cells, CD8+ T cells, neutrophils, macrophages and dendritic cells than those in low-risk group (all *P* < 0.05, Figure 6B).

Immune checkpoints have been the subject of a wave of new studies for their important roles in immune regulation, and immune checkpoint blockade therapies are promising strategies in the treatment of cancer (28). Therefore, we investigated the relationship between the IPS and expression of critical immune checkpoints including PD-1, CTLA-4, LAG-3, TIM-3, and TIGIT. We found that the risk score showed a positive correlation with the expression of PD-1, CTLA-4, TIM-3, and TIGIT (Figure 6C). Among the risk groups, high-risk patients expressed higher levels of CTLA-4, PD-1, and TIM-3 (all *P* < 0.05, Figure 6D, Supplementary Table 3).

### Functional Annotation of Prognostic DEIGs Between High- and Low-Risk Group

**Figure 7:**
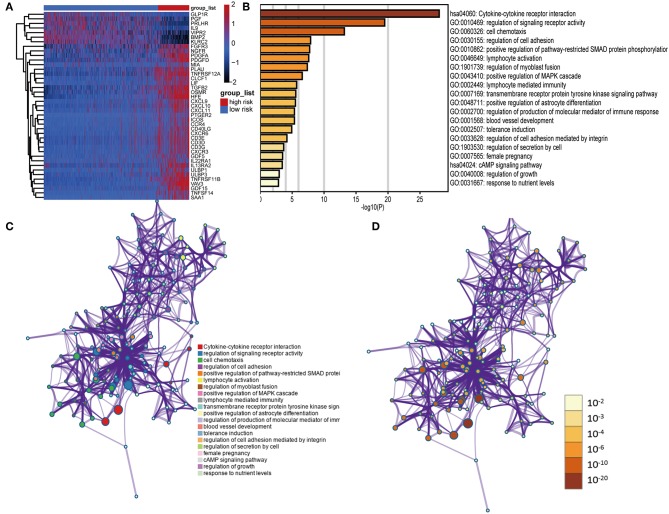
Functional analysis of 41 risk score-associated genes. **(A)** Heatmap of IDH1-associated immune genes that were differentially expressed between patients with high- and low-risk scores. **(B)** Heatmap of enriched terms across input gene lists, colored by *P*-values. Network of enriched terms: **(C)** colored by cluster ID, where nodes that share the same cluster ID are typically close to each other; **(D)** colored by *P*-value, where terms containing more genes tend to have a more significant *P*-value.

We identified 41 DEIGs validated in the CGGA database that were risk score-associated genes. These genes were differentially expressed between high- and low-risk LGG patients (Figure 7A). Similar to the results from the gene enrichment analysis of 88 DEIGs, these prognostic risk score-associated genes were mainly enriched in regulation of signaling receptor activity, cell chemotaxis, positive regulation of pathway-restricted SMAD protein phosphorylation, lymphocyte activation, positive regulation of MAPK cascade (GO), and cytokine-cytokine receptor interaction (KEGG, Figures 7B–D). This data thus provided a deeper understanding of the biological effects of the IPS.

### IPS Was an Independent Predictive Marker of OS for LGG Patients

**Figure 8:**
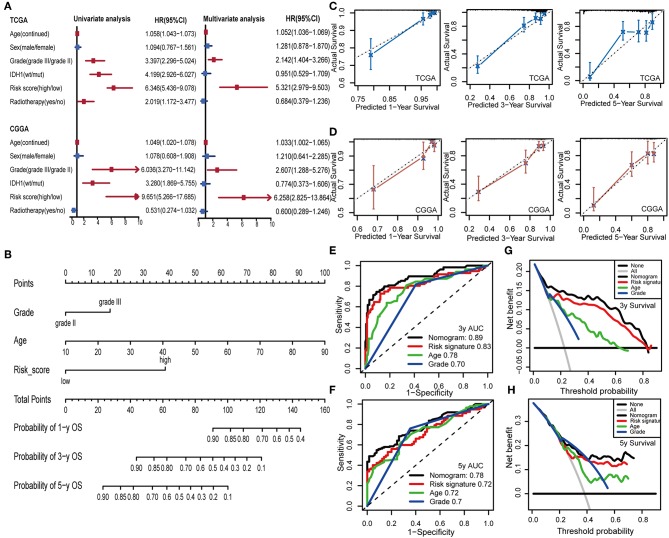
Construction and validation of the nomogram model. **(A)** Univariate and multivariate Cox analyses indicated that IPS was significantly associated with OS in both TCGA and CGGA sets. Red indicates statistical significance, and blue indicates no statistical significance. **(B)** Nomogram model for predicting the probability of 1-, 3-, and 5-year OS in LGGs. **(C,D)** Calibration plots of the nomogram for predicting the probability of OS at 1, 3, and 5 years in TCGA and CGGA cohorts. **(E,F)** Time-dependent ROC curve analyses of the nomogram model, risk signature, age and tumor grade in TCGA cohort. **(G,H)** Decision curves of the nomogram predicting 3- and 5-year OS in TCGA cohort.

To examine whether the IPS was an independent prognostic factor for LGG patients, we first applied univariate Cox analysis and found that the IPS was significantly associated with OS [Hazard ratio (HR): 6.346, 95% confidence interval (CI): 5.436–9.078, *P* < 0.001; Figure 8A]. By adjusting for the available clinicopathological variables, multivariate Cox analysis revealed that the IPS was able to serve as an independent prognostic factor with a HR of 5.321 in the TCGA cohort (95% CI: 2.979–9.503, *P* < 0.001; Figure 8A). In addition, the same results were found in the CGGA cohort and indicated that the IPS had an independent role in predicting LGG survival (univariate: HR: 9.651, 95% CI: 5.266–17.685, *P* < 0.001; multivariate: HR:6.258, 95% CI: 2.825–13.864, *P* < 0.001; Figure 8A).

### Establishment and Validation of an IPS-Based Nomogram Model

To provide a clinically associated quantitative method that could be employed to estimate OS for LGG patients, we developed a nomogram model in which the IPS integrated the two independent prognostic factors (age and grade; Figure 8B). The C-index values indicated favorable discrimination ability of the nomogram model (TCGA: C-index 0.839; CGGA: C-index 0.811). Calibration plots of observed vs. predicted probabilities of 1-, 3-, and 5-year OS demonstrated excellent concordance in both the TCGA (Figure 8C) and CGGA cohorts (Figure 8D). We then used time-dependent ROC curve analysis to compare the predictive accuracy between the nomogram model and individual predictors, including IPS, age, and grade (Figures 8E,F). The nomogram model suggested higher prognostic accuracy at 3-and 5-year OS with a larger AUC. Ultimately, we attempted to determine the clinical benefit of the nomogram model and the corresponding scope of application via DCA. Compared with IPS, age and tumor grade, the nomogram mode revealed an enhanced net benefit with wider threshold probabilities and offered the best clinical utility (3-year OS: Figure 8G; 5-year OS: Figure 8H).

## Discussion

Although many new molecular markers have been identified, the IDH1 mutation remains the most stable, and is widely used in glioma studies (29). The discovery of IDH mutations in gliomas as compared to their IDH wildtype counterparts, plays a crucial part in the understanding of glioma biology. Mounting evidence reveals that the immunological tumor microenvironment of the gliomas differs based on their IDH1 mutation (15). However, the mechanism governing the association of IDH1 mutation with the immune microenvironment is yet to be studied.

In the current study, the role of IDH1 mutations in the regulation of immune phenotype in LGGs was comprehensively studied. An IDH1-associated IPS, which was significantly related to prognosis, was constructed based on a TCGA set, and validated in a CGGA set. The prognostic value of this four-gene IPS was also independent of the known strong prognostic factors, like IDH1 mutation, age, and tumor grade. In addition, the IPS enabled us to classify patients into subgroups with distinct outcomes and immunophenotypes, implying that it may be used to refine the current prognostic model and facilitate further stratification of patients. Therefore, we leveraged the complementary value of molecular and clinical characteristics, and integrated them to develop a novel nomogram model to provide superior survival prediction. Further bioinformatics analysis was conducted to better understand the biological function of these IDH1-associated immune prognostic genes.

The four genes included in our signature were HFE, VAV3, TNFRSF12A, and TNFRSF11B. Notably, there is no overlap between the IDH1-associated immune genes identified in the aforementioned studies. Moreover, these selected genes hold great promise to serve as novel molecular targets and improve patient management in the era of immunotherapy. The HFE gene encodes the HFE protein, an MHC I-like molecule that acts as an iron sensor in the body and is involved in iron metabolism (30). There is increasing evidence suggesting a role for HFE in antigen presentation with interactions between HFE and the antigen presentation pathway shown to impair antigen processing and T cell activation (31, 32). Previous studies have also demonstrated a relationship between HFE genotype and increased frequency of cancer. In patients with diffuse gliomas, HFE expression was associated with decreased survival (33). VAV3, a Rho-GTPase guanine nucleotide exchange factor, is widely expressed in multiple tissues and plays important roles in the formation of the cytoskeleton, cell differentiation, regulation of T and B cell signaling pathways, and oncogenesis (34, 35). Liu et al. demonstrated that high expression of VAV3 was related to poor survival in glioblastomas (36), whereas its effect on LGG prognosis was not identified previously. Furthermore, TNFRSF12A and TNFRSF11B are cytokine receptors belonging to the tumor necrosis factor receptor superfamily. Weller et al. explored the association between TNFRSF11B and Apo2L/TRAIL-based therapy in gliomas (37), but the underlying mechanisms of its involvement in tumor biology remains to be investigated. In our study, elevated expression of VAV3 and TNFRSF11B were found to be related to worse survival in LGGs for the first time.

Characterization of the immune infiltration landscape is of great significance in investigation of the cross-talk between tumors and immunity. Thus, we explored the correlation between the IPS and immune cell infiltration to reflect the status of the immune microenvironment in LGGs. On basis of the TIMER database, we found that the high-risk patients had higher infiltrating levels of B cells, CD4+ T cells, CD8+ T cells, neutrophils, macrophages, and dendritic cells. These results confirmed and expanded the finding that the heterogeneity of immune infiltration was crucial for LGG progression. The IPS could be used as a predictor for increased immune cell infiltration and may have significant clinical implications.

Currently, there are an unprecedented number of clinical trials evaluating the effects of immune checkpoint inhibitors in gliomas (38). Further analysis was conducted to explore the association between IPS and the expression of critical immune checkpoints. We found that high-risk patients had higher PD-1, CTLA-4, and TIM-3 expression in the tumor microenvironment suggesting that the immunosuppressive microenvironment partly led to worse survival of these patients. Thus, these patients might be more likely to benefit from immune checkpoint blockade therapies.

The current study provided novel insights into the LGG immune microenvironment and immunotherapies. The selected genes should be prioritized for functional and mechanistic studies to confirm the value of their clinical application. Moreover, a limitation of this study is its retrospective nature. Thus, further prospective studies are needed.

In summary, the IPS is a clinically promising biomarker that can be used to classify LGG patients into subgroups with distinct outcomes and immunophenotypes, with the potential to facilitate individualized management and improve prognosis. It also provides a novel way to elucidate the mechanism of the IDH1 mutation on prognosis from an immunological perspective.

## Data Availability Statement

Publicly available datasets were analyzed in this study. This data can be found here: TCGA database (https://www.cancer.gov/).

## Author Contributions

XD and DL designed the study and wrote the initial draft of the manuscript. XD, BC, XZ, and XX contributed to data analysis. ZY, XS, LY, XL, HS, BY, NZ, and JL reviewed and edited the manuscript. All authors read and approved the manuscript.

### Conflict of Interest

The authors declare that the research was conducted in the absence of any commercial or financial relationships that could be construed as a potential conflict of interest.
