# Supplementary material for: Development and Validation of an IDH1-Associated Immune Prognostic Signature for Diffuse Lower-Grade Glioma
Source: Front Oncol. 2019 Nov 22;9:1310. doi: 10.3389/fonc.2019.01310 (PMC6883600; doi:10.3389/fonc.2019.01310)
Supplement: Supplementary file 1 [file Table_1.DOCX]

**Supplementary Table 1.** 41 IDH1-associated immune prognostic genes.

|  | Gene symbol |
| --- | --- |
| 1 | PRLHR |
| 2 | TNFRSF12A |
| 3 | TNFRSF11B |
| 4 | ULBP1 |
| 5 | CXCL10 |
| 6 | TGFB2 |
| 7 | IL9 |
| 8 | OSMR |
| 9 | VAV3 |
| 10 | CD3E |
| 11 | GDF15 |
| 12 | BMP2 |
| 13 | NGFR |
| 14 | ICOS |
| 15 | CD3G |
| 16 | CXCR6 |
| 17 | PLAU |
| 18 | CXCL9 |
| 19 | PDGFD |
| 20 | IL13RA2 |
| 21 | GLP1R |
| 22 | PTGER2 |
| 23 | GDF5 |
| 24 | CLCF1 |
| 25 | CD3D |
| 26 | ULBP3 |
| 27 | CXCL11 |
| 28 | PDGFA |
| 29 | TNFSF14 |
| 30 | MIA |
| 31 | FGFR3 |
| 32 | HFE |
| 33 | CCR4 |
| 34 | KLRC2 |
| 35 | CD40LG |
| 36 | LIF |
| 37 | PGF |
| 38 | CXCR3 |
| 39 | IL22RA1 |
| 40 | SAA1 |
| 41 | VIPR2 |
