# Supplementary material for: Development and Validation of an IDH1-Associated Immune Prognostic Signature for Diffuse Lower-Grade Glioma
Source: Front Oncol. 2019 Nov 22;9:1310. doi: 10.3389/fonc.2019.01310 (PMC6883600; doi:10.3389/fonc.2019.01310)
Supplement: Supplementary file 3 [file Table_3.DOCX]

**Supplementary Table 3.** Analysis of correlations between risk score and

immune checkpoints.

| **Variable 1** | **Variable 2** | **Pearson correlation coefficient** | **P value** |
| --- | --- | --- | --- |
| Risk score | PD-1 | 0.402287295 | 4.18E-21 |
| Risk score | CLAT-4 | 0.331735904 | 1.83E-14 |
| Risk score | TIM-3 | 0.408335206 | 9.38E-22 |
| Risk score | LAG-3 | 0.070559699 | 1.13E-01 |
| Risk score | TIGIT | 0.094330775 | 3.39E-02 |
| PD-1 | CLAT-4 | 0.41273096 | 3.11E-22 |
| PD-1 | TIM-3 | 0.460673797 | 6.00E-28 |
| PD-1 | LAG-3 | 0.430649689 | 2.90E-24 |
| PD-1 | TIGIT | 0.156313786 | 4.17E-04 |
| CLAT-4 | TIM-3 | 0.363352161 | 3.08E-17 |
| CLAT-4 | LAG-3 | 0.353908816 | 2.24E-16 |
| CLAT-4 | TIGIT | 0.253995027 | 6.85E-09 |
| TIM-3 | LAG-3 | 0.247580501 | 1.67E-08 |
| TIM-3 | TIGIT | 0.046485575 | 2.97E-01 |
| LAG-3 | TIGIT | 0.063326504 | 1.55E-01 |
